# EHEALTH LITERACY AMONG OLDER ADULTS: THE ROLE OF DIGITAL LITERACY AND SELF-EFFICACY

**DOI:** 10.1093/geroni/igae098.3408

**Published:** 2024-12-31

**Authors:** Linda Göbl, Mario R Jokisch, Hans-Werner Wahl, David Leopold, Michael Doh

**Affiliations:** Catholic University Freiburg, Freiburg im Breisgau, Baden-Wurttemberg, Germany; Kempten University of Applied Sciences, Freiburg im Breisgau, Baden-Wurttemberg, Germany; Heidelberg University, Heidelberg, Baden-Wurttemberg, Germany; Catholic University Freiburg, Freiburg im Breisgau, Baden-Wurttemberg, Germany; Catholic University Freiburg, Freiburg im Breisgau, Baden-Wurttemberg, Germany

## Abstract

Particularly for older adults with an increased risk of multimorbidity, digital health technologies can provide added value for health management. Successful use of these technologies requires appropriate skills. Key constructs are eHealth literacy and internet self-efficacy, which become particularly relevant in older age. This study investigated the relationship between these two multifactorial constructs in older adults, with eHealth literacy as a dependent variable, focusing on the mediating effect of digital literacy, and task-specific eHealth self-efficacy (i.e., contacting doctors via video communication and online health information seeking). A cross-sectional dataset with 327 internet-savvy German adults (mean age=71.75±6.06; 41% female) was analyzed using a structural equation model. After controlling for age, sex and subjective health, general internet self-efficacy showed a positive direct association on eHealth self-efficacy (β=.45, p< 0.01). General internet self-efficacy showed no indirect association on eHealth literacy, mediated by digital literacy, but a positive direct association on digital literacy (β=.72, p< 0.001). Digital literacy (β=.30, p< 0.05) was the strongest predictor of eHealth literacy. The model fit the data good (CFI=0.980; RMSEA=0.044) and explained 22.9% of the variance of eHealth literacy and 69.7% of the variance of digital literacy. These results point to the need for programs improving eHealth literacy to also focus on the use of digital devices and the promotion of general and task-specific internet self-efficacy. Implications for public health strategies will be discussed in order to improve eHealth literacy among older adults in a targeted manner and enable the successful use of digital health technologies.

